# Personal Mention

**Published:** 1915-11

**Authors:** 


### Personal Mention.

Dr. Gaither and family have moved to Glenrose.

Dr. O. B. Hicks of Atlanta has moved to Shreveport, La.

Dr. H. G. Shytles of Fort Worth has moved to Venus, Texas.

Dr. Pruit of Georges Creek has moved his family to Glenrose, Texas.

Dr. M. T. Griffin and family have moved from Seymour to Dallas.

Dr. and Mrs. A. Jacoby and family have moved from New Orleans, La., to Dallas, Texas.

Dr. T. J. Barrett, once editor of the *Hidalgo County Advance*, with his wife, has moved to Pharr.

Dr. J. H. Masey of Seadrift has returned from New Orleans, where he has been attending school.

Dr. S. T. Sherman and family have moved from Atlanta to Marshall to make their future home.

Dr. I. L. McGlasson of Waco has gone to Johns Hopkins in Baltimore to take a post-graduate course.

Dr. and Mrs. R. A. Maddox of Abilene have returned from an extended trip in Kansas and other points.

Dr. Joe H. Warnick and family have moved from Merkel to Dallas, Texas, where Dr. Warnick will engage in practice.

Dr. G. M. Duckworth of Cuero has returned from Chicago, where he has been taking post-graduate work in a hospital of that city.

Dr. W. Wilson of Memphis has gone to Boston, Mass., to attend the International Congress of Surgery. He will be in the East for a month.

Dr. H. F. Nugent, of Anson, who had both legs broken and crushed last winter, is able to resume his practice again. His many friends are very happy over his recovery.

Dr. Allen G. Heard, adjunct professor of medicine and diseases of children, addressed the medical department of the University of Texas at its opening exercises at Galveston in September.

Dr. T. G. Howe has bought out Drs. S. T. Sherman and O. B. Hicks, and he with his family will move to Atlanta from Douglassville soon. Dr. Una Howe, daughter of Dr. T. G. Howe, took charge of the office over the Morris Drug Co.

Dr. and Mrs. W. R. Jamieson of El Paso have gone for an extended visit through the North and East. They will go first to New York and then visit points of interest in New England and in Canada, returning to El Paso at the end of a month.

Dr. J. W. King of Bradford, Pa., recently told an interesting story of how he treated a negress for lumbago by freezing one leg from the knee down to the ankle with ether. He states that the frozen limb was perfectly white when he last saw the negress, some ten days after the treatment.

The many friends of Dr. J. S. Lankford are congratulating him on the very excellent article he recently contributed to the New York Medical Journal entitled "Life Insurance and the Kidneys." The article is not only valuable as a factor in life insurance risks, but it is instructive and interesting to the lay reader as well, and indicates a profundity in medical science that entitles the author to the distinction accorded him by the high class publication in which his article appeared.

